# First record of the genus *Venanus* (Hymenoptera: Braconidae: Microgastrinae) in Mesoamerica, with the description of two new species from Costa Rica

**DOI:** 10.3897/BDJ.2.e4167

**Published:** 2014-11-13

**Authors:** Jose L Fernandez-Triana, James B Whitfield, M. Alex Smith, Winnie Hallwachs, Daniel H. Janzen

**Affiliations:** † Canadian National Collection of Insects, Ottawa, and Biodiversity Institute of Ontario, University of Guelph, Ottawa, Canada; ‡ University of Illinois, Champaign, United States of America; § Department of Integrative Biology and the Biodiversity Institute of Ontario, Canada; | Department of Biology, University of Pennsylvania, Philadelphia, PA 19104, USQA, United States of America; ¶ Department of Biology, University of Pennsylvania, Philadelphia, United States of America

**Keywords:** *
Venanus
*, Microgastrinae, taxonomy, Area de Conservación Guancaste, Costa Rica, Mesoamerica

## Abstract

The New World genus *Venanus* (Hymenoptera: Braconidae: Microgastrinae) is a small group of parasitoid wasps that includes two Nearctic and seven Neotropical species. Here two additional species, authored by Fernández-Triana & Whitfield, are described from Costa Rica: *V. johnnyrosalesi* **sp. n.** from Area de Conservación Guanacaste (ACG) and *V. randallgarciai* **sp. n.** from Area de Conservación Cordillera Volcanica Central. They represent the first record of the genus for Mesoamerica. A previous key to all known *Venanus* (Whitfield et al. 2011) is modified to include the new species. The Costa Rican species were collected at altitudes of 1,400–1,460 m, but nothing is known of their biology. DNA barcodes were obtained for both species and are included as part of the description along with extensive photos. This paper is part of a series inventorying the diversity of Microgastrinae in ACG.

## Introduction

Microgastrine wasps (Hymenoptera: Braconidae) have been intensively studied for the past few years in Area de Conservación Guanacaste (ACG), northwestern Costa Rica (Whitfield et al. 2011, Fernández-Triana et al. 2014). ACG has been inventorying all caterpillar taxa, their food plants, and their parasitoids since 1978 (Janzen et al. 2009, Janzen et al. 2012, Fernández-Triana et al. 2014). That work provides some of the world’s largest tropical location-based datasets for studying the taxonomy and host relationships of caterpillar parasitoids.

This paper is a continuation of those efforts, and deals with the genus *Venanus* in ACG. An additional species from Costa Rica (but not from ACG) was found in the Canadian National Collection of Insects, Ottawa (CNC) and is also described below. The genus *Venanus* has been revised recently (Whitfield et al. 2011, Fernandez-Triana 2010) and nine species have been described from North and South America. The Costa Rican species represent the first record of *Venanus* for Mesoamerica.

## Materials and methods

*Venanus* is a rarely collected genus of Microgastrinae, and it is generally poorly represented in collections. This study is based on 22 specimens from the ACG inventory, and 1 specimen from Alajuela province (in Costa Rica but not in ACG) which was found in the CNC.

Morphological terms and measurements of structures are mostly as used by Mason (1981), Huber and Sharkey (1993), Whitfield (1997), Karlsson and Ronquist (2012), Fernández-Triana et al. (2014). The descriptions are brief and only include some body measurements that are commonly used in describing Microgastrinae (e.g., length of body, fore wing, and ovipositor sheath). These are complemented by extensive color photos of every species to illustrate, instead of describing with words, other details (e.g., color, shape, and sculpture). Geographic distribution is also provided in the key as supplementary information to aid the morphological identification of species.

Photos were taken with a Keyence VHX-1000 Digital Microscope, using a lens with a range of 13–130×. Multiple images through the focal plane were taken of a structure and these were combined to produce a single in-focus image, using the software associated with the Keyence System.

Detailed information about examined specimens (section "Materials" under Taxon treatments) was taken directly from the Barcode of Life Data System (BOLD) and imported into the Pensoft Writing Tool, as described in Fernandez-Triana et al. (2014).

In addition to the morphological descriptions, we provide DNA barcodes (the standardised region at the 5’ end of the cytochrome *c* oxidase I (CO1) gene, Hebert et al. 2003) whenever available. DNA barcodes for all ACG inventory specimens were obtained using DNA extracts prepared from single legs using a glass fibre protocol (Ivanova et al. 2006). Total genomic DNA was re-suspended in 30 μl of dH_2_O, and a 658-bp region near the 5’ terminus of the CO1 gene was amplified using standard primers (LepF1–LepR1) following established protocols (Smith et al. 2006, Smith et al. 2007, Smith et al. 2008). All information for the sequences associated with each individual specimen can be retrieved from BOLD (Ratnasingham and Hebert 2007) via the publically available dataset: http://dx.doi.org/10.5883/DS-VENANUS. A K2P tree was also generated with all sequences of described species of *Venanus* over 400 base pairs that were available in BOLD (Suppl. material 1).

## Taxon treatments

### Venanus

Mason, 1981

Venanus Mason, 1981: 94.

#### Diagnosis

The genus *Venanus* can be recognized by the following combination of features: Body shape shape relatively slender, often somewhat dorsoventrally flattened. Body color typically black nearly throughout, legs variable in color. Fore wing with closed areolet (r-m present). Metacoxae relatively small (as in *Microplitis*). Propodeum rugose, with medial carina present, at least for some portion of length. First metasomal tergite relatively elongate, of somewhat variable shape and degree of sculpturing. Second metasomal tergum with median raised area that is narrower than first tergite, at least at their junction. Ovipositor sheath distally with setae highly reduced in size (as in *Distatrix*, and *Venanides*). The genus is restricted to the New World, from as north as Canada (Yukon Territory) to Chile in South America (Mason 1981, Fernandez-Triana 2010, Whitfield et al. 2011). It is a relatively small genus, with nine species previoulsy described and a few other apparent new species found in collections (Whitfield et al. 2011). Leafmining and needle mining caterpillars were believed to be the main hosts (Mason 1981), however recently collecting and rearing of caterpiilas in South America suggests that other hosts such as Pyralidae might also be common (Whitfield et al. 2011).

### Venanus johnnyrosalesi

Fernández-Triana & Whitfield sp. n.

urn:lsid:zoobank.org:act:FF576CD7-7AEB-4F14-B001-3BD0985222FA

#### Materials

- **Type status:** Holotype. **Occurrence:** occurrenceDetails: http://janzen.sas.upenn.edu/caterpillars/database.lasso; catalogNumber: DHJPAR0031445; recordedBy: D.H.Janzen&W.Hallwachs; individualID: DHJPAR0031445; individualCount: 1; sex: Female; lifeStage: adult; otherCatalogNumbers: 08-SRNP-38355; **Taxon:** scientificName: Venanus johnnyrosalesi; phylum: Arthropoda; class: Insecta; order: Hymenoptera; family: Braconidae; genus: Venanus; specificEpithet: johnnyrosalesi; scientificNameAuthorship: Fernández-Triana; **Location:** continent: Central America; country: Costa Rica; stateProvince: Guanacaste; locality: Area de Conservacion Guanacaste; verbatimLocality: Sendero Cima; verbatimElevation: 1460 m; verbatimLatitude: 10.9333; verbatimLongitude: -85.4573; verbatimCoordinateSystem: Decimal; decimalLatitude: 10.9333; decimalLongitude: -85.4573; **Identification:** dateIdentified: 2014; **Event:** samplingProtocol: Malaise Trap; verbatimEventDate: 11/17/2008; **Record Level:** language: en; institutionCode: CNC; collectionCode: Insects; basisOfRecord: PreservedSpecimen
- **Type status:** Paratype. **Occurrence:** occurrenceDetails: http://janzen.sas.upenn.edu/caterpillars/database.lasso; catalogNumber: DHJPAR0031434; recordedBy: D.H.Janzen&W.Hallwachs; individualID: DHJPAR0031434; individualCount: 1; sex: Female; lifeStage: adult; otherCatalogNumbers: 08-SRNP-38344; **Taxon:** scientificName: Venanus johnnyrosalesi; phylum: Arthropoda; class: Insecta; order: Hymenoptera; family: Braconidae; genus: Venanus; specificEpithet: johnnyrosalesi; scientificNameAuthorship: Fernández-Triana; **Location:** continent: Central America; country: Costa Rica; stateProvince: Guanacaste; locality: Area de Conservacion Guanacaste; verbatimLocality: Sendero Cima; verbatimElevation: 1460 m; verbatimLatitude: 10.9333; verbatimLongitude: -85.4573; verbatimCoordinateSystem: Decimal; decimalLatitude: 10.9333; decimalLongitude: -85.4573; **Identification:** dateIdentified: 2014; **Event:** samplingProtocol: Malaise Trap; verbatimEventDate: 10/27/2008; **Record Level:** language: en; institutionCode: CNC; collectionCode: Insects; basisOfRecord: PreservedSpecimen
- **Type status:** Paratype. **Occurrence:** occurrenceDetails: http://janzen.sas.upenn.edu/caterpillars/database.lasso; catalogNumber: DHJPAR0031452; recordedBy: D.H.Janzen&W.Hallwachs; individualID: DHJPAR0031452; individualCount: 1; sex: Female; lifeStage: adult; otherCatalogNumbers: 08-SRNP-38362; **Taxon:** scientificName: Venanus johnnyrosalesi; phylum: Arthropoda; class: Insecta; order: Hymenoptera; family: Braconidae; genus: Venanus; specificEpithet: johnnyrosalesi; scientificNameAuthorship: Fernández-Triana; **Location:** continent: Central America; country: Costa Rica; stateProvince: Guanacaste; locality: Area de Conservacion Guanacaste; verbatimLocality: Sendero Cima; verbatimElevation: 1460 m; verbatimLatitude: 10.9333; verbatimLongitude: -85.4573; verbatimCoordinateSystem: Decimal; decimalLatitude: 10.9333; decimalLongitude: -85.4573; **Identification:** dateIdentified: 2014; **Event:** samplingProtocol: Malaise Trap; verbatimEventDate: 12/01/2008; **Record Level:** language: en; institutionCode: CNC; collectionCode: Insects; basisOfRecord: PreservedSpecimen
- **Type status:** Paratype. **Occurrence:** occurrenceDetails: http://janzen.sas.upenn.edu/caterpillars/database.lasso; catalogNumber: DHJPAR0031455; recordedBy: D.H.Janzen&W.Hallwachs; individualID: DHJPAR0031455; individualCount: 1; sex: Male; lifeStage: adult; otherCatalogNumbers: 08-SRNP-38365; **Taxon:** scientificName: Venanus johnnyrosalesi; phylum: Arthropoda; class: Insecta; order: Hymenoptera; family: Braconidae; genus: Venanus; specificEpithet: johnnyrosalesi; scientificNameAuthorship: Fernández-Triana; **Location:** continent: Central America; country: Costa Rica; stateProvince: Guanacaste; locality: Area de Conservacion Guanacaste; verbatimLocality: Sendero Cima; verbatimElevation: 1460 m; verbatimLatitude: 10.9333; verbatimLongitude: -85.4573; verbatimCoordinateSystem: Decimal; decimalLatitude: 10.9333; decimalLongitude: -85.4573; **Identification:** dateIdentified: 2014; **Event:** samplingProtocol: Malaise Trap; verbatimEventDate: 12/15/2008; **Record Level:** language: en; institutionCode: CNC; collectionCode: Insects; basisOfRecord: PreservedSpecimen
- **Type status:** Paratype. **Occurrence:** occurrenceDetails: http://janzen.sas.upenn.edu/caterpillars/database.lasso; catalogNumber: DHJPAR0031456; recordedBy: D.H.Janzen&W.Hallwachs; individualID: DHJPAR0031456; individualCount: 1; sex: Male; lifeStage: adult; otherCatalogNumbers: 08-SRNP-38366; **Taxon:** scientificName: Venanus johnnyrosalesi; phylum: Arthropoda; class: Insecta; order: Hymenoptera; family: Braconidae; genus: Venanus; specificEpithet: johnnyrosalesi; scientificNameAuthorship: Fernández-Triana; **Location:** continent: Central America; country: Costa Rica; stateProvince: Guanacaste; locality: Area de Conservacion Guanacaste; verbatimLocality: Sendero Cima; verbatimElevation: 1460 m; verbatimLatitude: 10.9333; verbatimLongitude: -85.4573; verbatimCoordinateSystem: Decimal; decimalLatitude: 10.9333; decimalLongitude: -85.4573; **Identification:** dateIdentified: 2014; **Event:** samplingProtocol: Malaise Trap; verbatimEventDate: 12/15/2008; **Record Level:** language: en; institutionCode: CNC; collectionCode: Insects; basisOfRecord: PreservedSpecimen
- **Type status:** Paratype. **Occurrence:** occurrenceDetails: http://janzen.sas.upenn.edu/caterpillars/database.lasso; catalogNumber: DHJPAR0031461; recordedBy: D.H.Janzen&W.Hallwachs; individualID: DHJPAR0031461; individualCount: 1; sex: Male; lifeStage: adult; otherCatalogNumbers: 08-SRNP-38371; **Taxon:** scientificName: Venanus johnnyrosalesi; phylum: Arthropoda; class: Insecta; order: Hymenoptera; family: Braconidae; genus: Venanus; specificEpithet: johnnyrosalesi; scientificNameAuthorship: Fernández-Triana; **Location:** continent: Central America; country: Costa Rica; stateProvince: Guanacaste; locality: Area de Conservacion Guanacaste; verbatimLocality: Sendero Cima; verbatimElevation: 1460 m; verbatimLatitude: 10.9333; verbatimLongitude: -85.4573; verbatimCoordinateSystem: Decimal; decimalLatitude: 10.9333; decimalLongitude: -85.4573; **Identification:** dateIdentified: 2014; **Event:** samplingProtocol: Malaise Trap; verbatimEventDate: 12/22/2008; **Record Level:** language: en; institutionCode: CNC; collectionCode: Insects; basisOfRecord: PreservedSpecimen
- **Type status:** Paratype. **Occurrence:** occurrenceDetails: http://janzen.sas.upenn.edu/caterpillars/database.lasso; catalogNumber: DHJPAR0031462; recordedBy: D.H.Janzen&W.Hallwachs; individualID: DHJPAR0031462; individualCount: 1; sex: Male; lifeStage: adult; otherCatalogNumbers: 08-SRNP-38372; **Taxon:** scientificName: Venanus johnnyrosalesi; phylum: Arthropoda; class: Insecta; order: Hymenoptera; family: Braconidae; genus: Venanus; specificEpithet: johnnyrosalesi; scientificNameAuthorship: Fernández-Triana; **Location:** continent: Central America; country: Costa Rica; stateProvince: Guanacaste; locality: Area de Conservacion Guanacaste; verbatimLocality: Sendero Cima; verbatimElevation: 1460 m; verbatimLatitude: 10.9333; verbatimLongitude: -85.4573; verbatimCoordinateSystem: Decimal; decimalLatitude: 10.9333; decimalLongitude: -85.4573; **Identification:** dateIdentified: 2014; **Event:** samplingProtocol: Malaise Trap; verbatimEventDate: 12/22/2008; **Record Level:** language: en; institutionCode: CNC; collectionCode: Insects; basisOfRecord: PreservedSpecimen
- **Type status:** Paratype. **Occurrence:** occurrenceDetails: http://janzen.sas.upenn.edu/caterpillars/database.lasso; catalogNumber: DHJPAR0031466; recordedBy: D.H.Janzen&W.Hallwachs; individualID: DHJPAR0031466; individualCount: 1; sex: Female; lifeStage: adult; otherCatalogNumbers: 08-SRNP-38376; **Taxon:** scientificName: Venanus johnnyrosalesi; phylum: Arthropoda; class: Insecta; order: Hymenoptera; family: Braconidae; genus: Venanus; specificEpithet: johnnyrosalesi; scientificNameAuthorship: Fernández-Triana; **Location:** continent: Central America; country: Costa Rica; stateProvince: Guanacaste; locality: Area de Conservacion Guanacaste; verbatimLocality: Sendero Cima; verbatimElevation: 1460 m; verbatimLatitude: 10.9333; verbatimLongitude: -85.4573; verbatimCoordinateSystem: Decimal; decimalLatitude: 10.9333; decimalLongitude: -85.4573; **Identification:** dateIdentified: 2014; **Event:** samplingProtocol: Malaise Trap; verbatimEventDate: 12/29/2008; **Record Level:** language: en; institutionCode: CNC; collectionCode: Insects; basisOfRecord: PreservedSpecimen
- **Type status:** Paratype. **Occurrence:** occurrenceDetails: http://janzen.sas.upenn.edu/caterpillars/database.lasso; catalogNumber: DHJPAR0031470; recordedBy: D.H.Janzen&W.Hallwachs; individualID: DHJPAR0031470; individualCount: 1; sex: Male; lifeStage: adult; otherCatalogNumbers: 08-SRNP-38380; **Taxon:** scientificName: Venanus johnnyrosalesi; phylum: Arthropoda; class: Insecta; order: Hymenoptera; family: Braconidae; genus: Venanus; specificEpithet: johnnyrosalesi; scientificNameAuthorship: Fernández-Triana; **Location:** continent: Central America; country: Costa Rica; stateProvince: Guanacaste; locality: Area de Conservacion Guanacaste; verbatimLocality: Sendero Cima; verbatimElevation: 1460 m; verbatimLatitude: 10.9333; verbatimLongitude: -85.4573; verbatimCoordinateSystem: Decimal; decimalLatitude: 10.9333; decimalLongitude: -85.4573; **Identification:** dateIdentified: 2014; **Event:** samplingProtocol: Malaise Trap; verbatimEventDate: 01/05/2009; **Record Level:** language: en; institutionCode: CNC; collectionCode: Insects; basisOfRecord: PreservedSpecimen
- **Type status:** Paratype. **Occurrence:** occurrenceDetails: http://janzen.sas.upenn.edu/caterpillars/database.lasso; catalogNumber: DHJPAR0031472; recordedBy: D.H.Janzen&W.Hallwachs; individualID: DHJPAR0031472; individualCount: 1; sex: Male; lifeStage: adult; otherCatalogNumbers: 08-SRNP-38382; **Taxon:** scientificName: Venanus johnnyrosalesi; phylum: Arthropoda; class: Insecta; order: Hymenoptera; family: Braconidae; genus: Venanus; specificEpithet: johnnyrosalesi; scientificNameAuthorship: Fernández-Triana; **Location:** continent: Central America; country: Costa Rica; stateProvince: Guanacaste; locality: Area de Conservacion Guanacaste; verbatimLocality: Sendero Cima; verbatimElevation: 1460 m; verbatimLatitude: 10.9333; verbatimLongitude: -85.4573; verbatimCoordinateSystem: Decimal; decimalLatitude: 10.9333; decimalLongitude: -85.4573; **Identification:** dateIdentified: 2014; **Event:** samplingProtocol: Malaise Trap; verbatimEventDate: 01/12/2009; **Record Level:** language: en; institutionCode: CNC; collectionCode: Insects; basisOfRecord: PreservedSpecimen
- **Type status:** Paratype. **Occurrence:** occurrenceDetails: http://janzen.sas.upenn.edu/caterpillars/database.lasso; catalogNumber: DHJPAR0031473; recordedBy: D.H.Janzen&W.Hallwachs; individualID: DHJPAR0031473; individualCount: 1; sex: Male; lifeStage: adult; otherCatalogNumbers: 08-SRNP-38383; **Taxon:** scientificName: Venanus johnnyrosalesi; phylum: Arthropoda; class: Insecta; order: Hymenoptera; family: Braconidae; genus: Venanus; specificEpithet: johnnyrosalesi; scientificNameAuthorship: Fernández-Triana; **Location:** continent: Central America; country: Costa Rica; stateProvince: Guanacaste; locality: Area de Conservacion Guanacaste; verbatimLocality: Sendero Cima; verbatimElevation: 1460 m; verbatimLatitude: 10.9333; verbatimLongitude: -85.4573; verbatimCoordinateSystem: Decimal; decimalLatitude: 10.9333; decimalLongitude: -85.4573; **Identification:** dateIdentified: 2014; **Event:** samplingProtocol: Malaise Trap; verbatimEventDate: 01/12/2009; **Record Level:** language: en; institutionCode: CNC; collectionCode: Insects; basisOfRecord: PreservedSpecimen
- **Type status:** Paratype. **Occurrence:** occurrenceDetails: http://janzen.sas.upenn.edu/caterpillars/database.lasso; catalogNumber: DHJPAR0031480; recordedBy: D.H.Janzen&W.Hallwachs; individualID: DHJPAR0031480; individualCount: 1; sex: Male; lifeStage: adult; otherCatalogNumbers: 08-SRNP-38390; **Taxon:** scientificName: Venanus johnnyrosalesi; phylum: Arthropoda; class: Insecta; order: Hymenoptera; family: Braconidae; genus: Venanus; specificEpithet: johnnyrosalesi; scientificNameAuthorship: Fernández-Triana; **Location:** continent: Central America; country: Costa Rica; stateProvince: Guanacaste; locality: Area de Conservacion Guanacaste; verbatimLocality: Sendero Cima; verbatimElevation: 1460 m; verbatimLatitude: 10.9333; verbatimLongitude: -85.4573; verbatimCoordinateSystem: Decimal; decimalLatitude: 10.9333; decimalLongitude: -85.4573; **Identification:** dateIdentified: 2014; **Event:** samplingProtocol: Malaise Trap; verbatimEventDate: 01/26/2009; **Record Level:** language: en; institutionCode: CNC; collectionCode: Insects; basisOfRecord: PreservedSpecimen
- **Type status:** Paratype. **Occurrence:** occurrenceDetails: http://janzen.sas.upenn.edu/caterpillars/database.lasso; catalogNumber: DHJPAR0031481; recordedBy: D.H.Janzen&W.Hallwachs; individualID: DHJPAR0031481; individualCount: 1; sex: Male; lifeStage: adult; otherCatalogNumbers: 08-SRNP-38391; **Taxon:** scientificName: Venanus johnnyrosalesi; phylum: Arthropoda; class: Insecta; order: Hymenoptera; family: Braconidae; genus: Venanus; specificEpithet: johnnyrosalesi; scientificNameAuthorship: Fernández-Triana; **Location:** continent: Central America; country: Costa Rica; stateProvince: Guanacaste; locality: Area de Conservacion Guanacaste; verbatimLocality: Sendero Cima; verbatimElevation: 1460 m; verbatimLatitude: 10.9333; verbatimLongitude: -85.4573; verbatimCoordinateSystem: Decimal; decimalLatitude: 10.9333; decimalLongitude: -85.4573; **Identification:** dateIdentified: 2014; **Event:** samplingProtocol: Malaise Trap; verbatimEventDate: 02/02/2009; **Record Level:** language: en; institutionCode: CNC; collectionCode: Insects; basisOfRecord: PreservedSpecimen
- **Type status:** Paratype. **Occurrence:** occurrenceDetails: http://janzen.sas.upenn.edu/caterpillars/database.lasso; catalogNumber: DHJPAR0031486; recordedBy: D.H.Janzen&W.Hallwachs; individualID: DHJPAR0031486; individualCount: 1; sex: Male; lifeStage: adult; otherCatalogNumbers: 08-SRNP-38396; **Taxon:** scientificName: Venanus johnnyrosalesi; phylum: Arthropoda; class: Insecta; order: Hymenoptera; family: Braconidae; genus: Venanus; specificEpithet: johnnyrosalesi; scientificNameAuthorship: Fernández-Triana; **Location:** continent: Central America; country: Costa Rica; stateProvince: Guanacaste; locality: Area de Conservacion Guanacaste; verbatimLocality: Sendero Cima; verbatimElevation: 1460 m; verbatimLatitude: 10.9333; verbatimLongitude: -85.4573; verbatimCoordinateSystem: Decimal; decimalLatitude: 10.9333; decimalLongitude: -85.4573; **Identification:** dateIdentified: 2014; **Event:** samplingProtocol: Malaise Trap; verbatimEventDate: 03/16/2009; **Record Level:** language: en; institutionCode: CNC; collectionCode: Insects; basisOfRecord: PreservedSpecimen
- **Type status:** Paratype. **Occurrence:** occurrenceDetails: http://janzen.sas.upenn.edu/caterpillars/database.lasso; catalogNumber: DHJPAR0031487; recordedBy: D.H.Janzen&W.Hallwachs; individualID: DHJPAR0031487; individualCount: 1; sex: Male; lifeStage: adult; otherCatalogNumbers: 08-SRNP-38397; **Taxon:** scientificName: Venanus johnnyrosalesi; phylum: Arthropoda; class: Insecta; order: Hymenoptera; family: Braconidae; genus: Venanus; specificEpithet: johnnyrosalesi; scientificNameAuthorship: Fernández-Triana; **Location:** continent: Central America; country: Costa Rica; stateProvince: Guanacaste; locality: Area de Conservacion Guanacaste; verbatimLocality: Sendero Cima; verbatimElevation: 1460 m; verbatimLatitude: 10.9333; verbatimLongitude: -85.4573; verbatimCoordinateSystem: Decimal; decimalLatitude: 10.9333; decimalLongitude: -85.4573; **Identification:** dateIdentified: 2014; **Event:** samplingProtocol: Malaise Trap; verbatimEventDate: 03/16/2009; **Record Level:** language: en; institutionCode: CNC; collectionCode: Insects; basisOfRecord: PreservedSpecimen
- **Type status:** Paratype. **Occurrence:** occurrenceDetails: http://janzen.sas.upenn.edu/caterpillars/database.lasso; catalogNumber: DHJPAR0031489; recordedBy: D.H.Janzen&W.Hallwachs; individualID: DHJPAR0031489; individualCount: 1; sex: Male; lifeStage: adult; otherCatalogNumbers: 08-SRNP-38399; **Taxon:** scientificName: Venanus johnnyrosalesi; phylum: Arthropoda; class: Insecta; order: Hymenoptera; family: Braconidae; genus: Venanus; specificEpithet: johnnyrosalesi; scientificNameAuthorship: Fernández-Triana; **Location:** continent: Central America; country: Costa Rica; stateProvince: Guanacaste; locality: Area de Conservacion Guanacaste; verbatimLocality: Sendero Cima; verbatimElevation: 1460 m; verbatimLatitude: 10.9333; verbatimLongitude: -85.4573; verbatimCoordinateSystem: Decimal; decimalLatitude: 10.9333; decimalLongitude: -85.4573; **Identification:** dateIdentified: 2014; **Event:** samplingProtocol: Malaise Trap; verbatimEventDate: 03/23/2009; **Record Level:** language: en; institutionCode: CNC; collectionCode: Insects; basisOfRecord: PreservedSpecimen
- **Type status:** Paratype. **Occurrence:** occurrenceDetails: http://janzen.sas.upenn.edu/caterpillars/database.lasso; catalogNumber: DHJPAR0031491; recordedBy: D.H.Janzen&W.Hallwachs; individualID: DHJPAR0031491; individualCount: 1; sex: Male; lifeStage: adult; otherCatalogNumbers: 08-SRNP-38401; **Taxon:** scientificName: Venanus johnnyrosalesi; phylum: Arthropoda; class: Insecta; order: Hymenoptera; family: Braconidae; genus: Venanus; specificEpithet: johnnyrosalesi; scientificNameAuthorship: Fernández-Triana; **Location:** continent: Central America; country: Costa Rica; stateProvince: Guanacaste; locality: Area de Conservacion Guanacaste; verbatimLocality: Sendero Cima; verbatimElevation: 1460 m; verbatimLatitude: 10.9333; verbatimLongitude: -85.4573; verbatimCoordinateSystem: Decimal; decimalLatitude: 10.9333; decimalLongitude: -85.4573; **Identification:** dateIdentified: 2014; **Event:** samplingProtocol: Malaise Trap; verbatimEventDate: 03/23/2009; **Record Level:** language: en; institutionCode: CNC; collectionCode: Insects; basisOfRecord: PreservedSpecimen
- **Type status:** Paratype. **Occurrence:** occurrenceDetails: http://janzen.sas.upenn.edu/caterpillars/database.lasso; catalogNumber: DHJPAR0031493; recordedBy: D.H.Janzen&W.Hallwachs; individualID: DHJPAR0031493; individualCount: 1; sex: Male; lifeStage: adult; otherCatalogNumbers: 08-SRNP-38403; **Taxon:** scientificName: Venanus johnnyrosalesi; phylum: Arthropoda; class: Insecta; order: Hymenoptera; family: Braconidae; genus: Venanus; specificEpithet: johnnyrosalesi; scientificNameAuthorship: Fernández-Triana; **Location:** continent: Central America; country: Costa Rica; stateProvince: Guanacaste; locality: Area de Conservacion Guanacaste; verbatimLocality: Sendero Cima; verbatimElevation: 1460 m; verbatimLatitude: 10.9333; verbatimLongitude: -85.4573; verbatimCoordinateSystem: Decimal; decimalLatitude: 10.9333; decimalLongitude: -85.4573; **Identification:** dateIdentified: 2014; **Event:** samplingProtocol: Malaise Trap; verbatimEventDate: 03/30/2009; **Record Level:** language: en; institutionCode: CNC; collectionCode: Insects; basisOfRecord: PreservedSpecimen
- **Type status:** Paratype. **Occurrence:** occurrenceDetails: http://janzen.sas.upenn.edu/caterpillars/database.lasso; catalogNumber: DHJPAR0031494; recordedBy: D.H.Janzen&W.Hallwachs; individualID: DHJPAR0031494; individualCount: 1; sex: Male; lifeStage: adult; otherCatalogNumbers: 08-SRNP-38404; **Taxon:** scientificName: Venanus johnnyrosalesi; phylum: Arthropoda; class: Insecta; order: Hymenoptera; family: Braconidae; genus: Venanus; specificEpithet: johnnyrosalesi; scientificNameAuthorship: Fernández-Triana; **Location:** continent: Central America; country: Costa Rica; stateProvince: Guanacaste; locality: Area de Conservacion Guanacaste; verbatimLocality: Sendero Cima; verbatimElevation: 1460 m; verbatimLatitude: 10.9333; verbatimLongitude: -85.4573; verbatimCoordinateSystem: Decimal; decimalLatitude: 10.9333; decimalLongitude: -85.4573; **Identification:** dateIdentified: 2014; **Event:** samplingProtocol: Malaise Trap; verbatimEventDate: 03/30/2009; **Record Level:** language: en; institutionCode: CNC; collectionCode: Insects; basisOfRecord: PreservedSpecimen
- **Type status:** Paratype. **Occurrence:** occurrenceDetails: http://janzen.sas.upenn.edu/caterpillars/database.lasso; catalogNumber: DHJPAR0031497; recordedBy: D.H.Janzen&W.Hallwachs; individualID: DHJPAR0031497; individualCount: 1; sex: Male; lifeStage: adult; otherCatalogNumbers: 08-SRNP-38407; **Taxon:** scientificName: Venanus johnnyrosalesi; phylum: Arthropoda; class: Insecta; order: Hymenoptera; family: Braconidae; genus: Venanus; specificEpithet: johnnyrosalesi; scientificNameAuthorship: Fernández-Triana; **Location:** continent: Central America; country: Costa Rica; stateProvince: Guanacaste; locality: Area de Conservacion Guanacaste; verbatimLocality: Sendero Cima; verbatimElevation: 1460 m; verbatimLatitude: 10.9333; verbatimLongitude: -85.4573; verbatimCoordinateSystem: Decimal; decimalLatitude: 10.9333; decimalLongitude: -85.4573; **Identification:** dateIdentified: 2014; **Event:** samplingProtocol: Malaise Trap; verbatimEventDate: 03/02/2009; **Record Level:** language: en; institutionCode: CNC; collectionCode: Insects; basisOfRecord: PreservedSpecimen
- **Type status:** Paratype. **Occurrence:** occurrenceDetails: http://janzen.sas.upenn.edu/caterpillars/database.lasso; catalogNumber: DHJPAR0031498; recordedBy: D.H.Janzen&W.Hallwachs; individualID: DHJPAR0031498; individualCount: 1; sex: Male; lifeStage: adult; otherCatalogNumbers: 08-SRNP-38408; **Taxon:** scientificName: Venanus johnnyrosalesi; phylum: Arthropoda; class: Insecta; order: Hymenoptera; family: Braconidae; genus: Venanus; specificEpithet: johnnyrosalesi; scientificNameAuthorship: Fernández-Triana; **Location:** continent: Central America; country: Costa Rica; stateProvince: Guanacaste; locality: Area de Conservacion Guanacaste; verbatimLocality: Sendero Cima; verbatimElevation: 1460 m; verbatimLatitude: 10.9333; verbatimLongitude: -85.4573; verbatimCoordinateSystem: Decimal; decimalLatitude: 10.9333; decimalLongitude: -85.4573; **Identification:** dateIdentified: 2014; **Event:** samplingProtocol: Malaise Trap; verbatimEventDate: 03/02/2009; **Record Level:** language: en; institutionCode: CNC; collectionCode: Insects; basisOfRecord: PreservedSpecimen
- **Type status:** Paratype. **Occurrence:** occurrenceDetails: http://janzen.sas.upenn.edu/caterpillars/database.lasso; catalogNumber: DHJPAR0031499; recordedBy: D.H.Janzen&W.Hallwachs; individualID: DHJPAR0031499; individualCount: 1; sex: Male; lifeStage: adult; otherCatalogNumbers: 08-SRNP-38409; **Taxon:** scientificName: Venanus johnnyrosalesi; phylum: Arthropoda; class: Insecta; order: Hymenoptera; family: Braconidae; genus: Venanus; specificEpithet: johnnyrosalesi; scientificNameAuthorship: Fernández-Triana; **Location:** continent: Central America; country: Costa Rica; stateProvince: Guanacaste; locality: Area de Conservacion Guanacaste; verbatimLocality: Sendero Cima; verbatimElevation: 1460 m; verbatimLatitude: 10.9333; verbatimLongitude: -85.4573; verbatimCoordinateSystem: Decimal; decimalLatitude: 10.9333; decimalLongitude: -85.4573; **Identification:** dateIdentified: 2014; **Event:** samplingProtocol: Malaise Trap; verbatimEventDate: 04/06/2009; **Record Level:** language: en; institutionCode: CNC; collectionCode: Insects; basisOfRecord: PreservedSpecimen

#### Description

**Figure 1.**
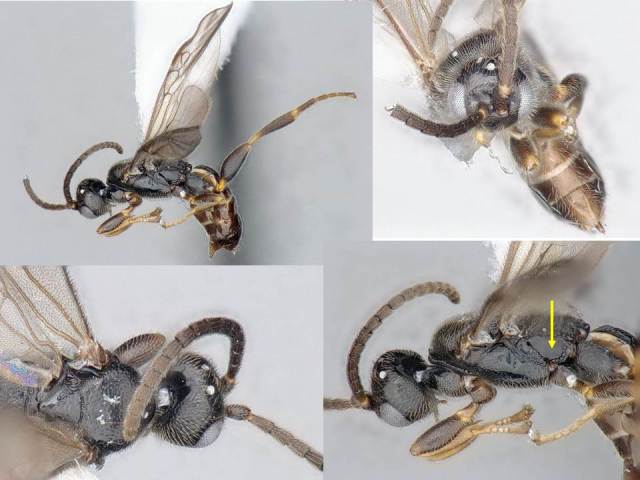
*Venanus johnnyrosalesi*. Yellow arrow shows the sculpture on metapleuron.

**Figure 2.**
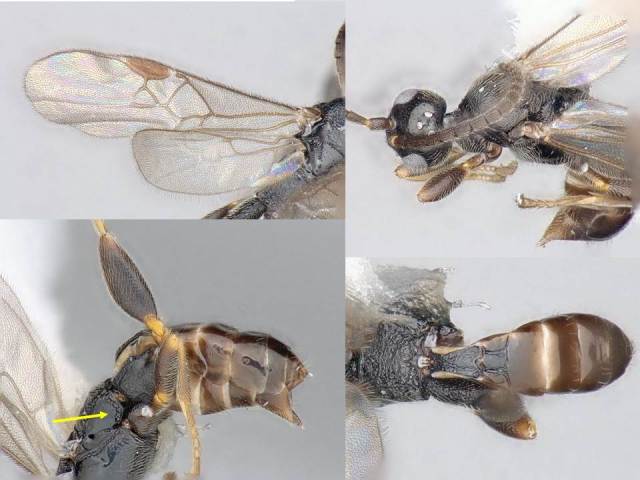
*Venanus johnnyrosalesi*. Yellow arrow shows the sculpture on metapleuron.

Female. Body length: 2.3 mm. Fore wing length: 2.2 mm. Flagellomere 2 length/width: 0.11 mm/0.05 mm. Flagellomere 14 length/width: 0.08 mm/0.06 mm. Oculo-ocellar distance: 0.14 mm. Distance between posterior ocelli: 0.08 mm. Diameter of posterior ocellus: 0.05 mm. Metafemur length/width: 0.52 mm/0.20 mm. Metatibia length: 0.62 mm. Mediotergite 1 length/maximum width/minimum width: 0.30/0.15/0.08 mm. Mediotergite 2 length/width at posterior margin: 0.13/0.08 mm. Figs 1, 2.

Male: As female, but metafemur thinner and antenna longer.

#### Diagnosis

The mediotergite 1 is relatively long and with a slight constriction near anterior end (Fig. 2). That character is also shared with other three species of *Venanus*. However, *V. johnnyrosalesi* can be separated from *V. helavai* by its much smaller size (2.3 mm vs 2.8–3.0 mm) and less sculptured propodeum, from *V. yanayacuensis* by its wider discal cell in fore wing (1.0 × vs 1.2 × as wide as high), metasoma color (brown vs black) and fore wing vein 2RS significanly longer than vein r (shorter than r in *yanayacuensis*), and from *V. randallgarciai* by proportion of veins 2RS and r (1.4 × vs 2.0 ×), sculpture of metapleuron and mediotergite 2, and less narrow mediotergite 1 (narrowest width 0.8 × width at posterior margin vs 0.6 × in *randallgarciai*).

#### Etymology

*Venanus johnnyrosalesi* is named in honor of Sr. Johnny Rosales, currently of San Jose, Costa Rica, but also a major user, appreciator and former director of ACG.

#### Distribution

Only know from Volcán Cacao, ACG, Costa Rica.

#### Notes

A total of 60 specimens (some of them not examined for this paper) were sampled for DNA, and 50 rendered full barcode sequences of 658 base pairs (see also Suppl. material 1). These sequences were characterised by very limited variation (a single synonymous, third base G/A transition). The holotype specimen (DHJPAR0031445) has the sequence accession ASHYG706-10 in BOLD (www.boldsystems.org) and the nucleotide sequence is reproduced below:

AATATTATACTTTATTTTTGGGTTATGAGCTGGTATAGTAGGATTTTCTATAAGAATAATCATTCGCTTAGAATTAGGAATACCTGGAAATTTAATTGGAAATGACCAAATTTATAATAGAATTGTTACTTCTCATGCTTTTATTATAATTTTTTTCATAGTTATACCAATCATAATTGGTGGATTTGGTAACTGATTAATTCCTTTAATATTAGGTACTCCAGATATAGCATTCCCTCGAATAAATAATATAAGATTTTGGTTACTTCTACCTTCATTATTTTTATTAATTTTAAGTAGATTTATTAATACAGGGGTAGGAACGGGATGAACAGTATACCCTCCTTTGTCATTAATTTTAGGCCATGGGGGAATATCAGTAGACCTGGGTATTTTTTCTCTTCATTTAGCAGGAATATCTTCAATTATAGGGGCTATTAATTTTATTTCCACAATTATAAATATACGAACAAATTTTTTAATAATAGACAAAATCTCTTTATTTTCATGATCTGTTTTAATTACAGCTATTTTATTACTTCTATCTTTACCAGTTTTAGCTGGAGCAATTACTATACTACTGACAGATCGAAATTTAAATACAAGATTTTTTGATCCAAGTGGAGGTGGAGATCCAATTCTTTATCAACATTTATTT

### Venanus randallgarciai

Fernández-Triana & Whitfield sp. n.

urn:lsid:zoobank.org:act:874E5AA8-587B-4FA4-835C-B5A3523FB554

#### Materials

- **Type status:** Holotype. **Occurrence:** catalogNumber: CNCHYM 07223; recordedBy: J. Helava; individualID: CNCHYM 07223; individualCount: 1; sex: female; lifeStage: adult; **Taxon:** scientificName: Venanus randallgarciai; phylum: Arthropoda; class: Insecta; order: Hymenoptera; family: Braconidae; genus: Venanus; specificEpithet: randallgarciai; scientificNameAuthorship: Fernández-Triana; **Location:** continent: Central America; country: Costa Rica; stateProvince: Alajuela; locality: 500 m North of Colonia Virgen del Socorro, Area de Conservacion Cordillera Volcanica Central; verbatimElevation: 1400 m; verbatimLatitude: 10° 17' N; verbatimLongitude: 84° 10' W; verbatimCoordinateSystem: Degree, minutes; **Identification:** dateIdentified: 2014; **Event:** verbatimEventDate: 30-v-1973; **Record Level:** language: en; collectionCode: Insects; ownerInstitutionCode: CNC; basisOfRecord: PreservedSpecimen

#### Description

**Figure 3.**
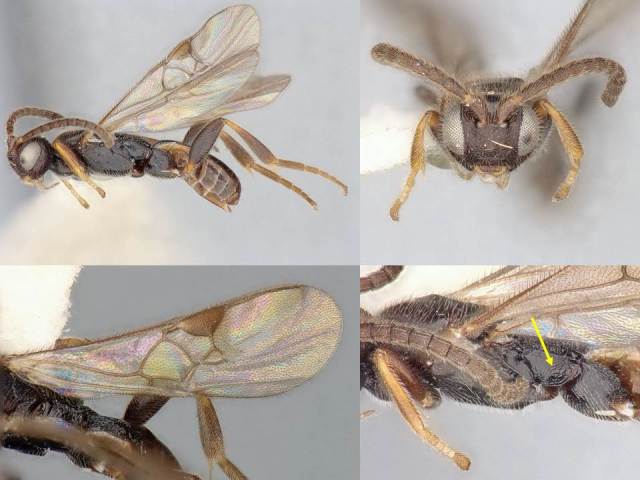
*Venanus randallgarciai*. Yellow arrow shows the sculpture on metapleuron.

**Figure 4.**
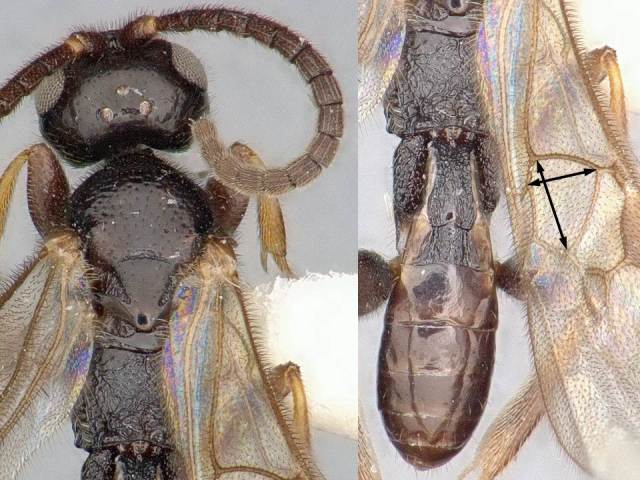
*Venanus randallgarciai*. Black arrows show length and width of first discal cell.

Female. Body length: 2.2 mm. Fore wing length: 2.2 mm. Flagellomere 2 length/width: 0.11 mm/0.06 mm. Flagellomere 14: missing. Oculo-ocellar distance: 0.15 mm. Distance between posterior ocelli: 0.08 mm. Diameter of posterior ocellus: 0.05 mm. Metafemur length/width: 0.53 mm/0.18 mm. Metatibia length: 0.66 mm. Mediotergite 1 length/maximum width/minimum width: 0.30/0.15/0.09 mm. Mediotergite 2 length/width at posterior margin: 0.12/0.10 mm. Figs 3, 4.

Male. Unknown.

#### Diagnosis

The mediotergite 1 is relatively long and with a slight constriction near anterior end (Fig. 4). That character is also shared with other three species of *Venanus*. However, *V. johnnyrosalesi* can be separated from *V. helavai* by its much smaller size (2.2 mm vs 2.8–3.0 mm) and less sculptured propodeum, from *V. yanayacuensis* by its wider discal cell in fore wing (1.0 × vs 1.2 × as wide as high), metasoma color (brown vs black) and fore wing vein 2RS significanly longer than vein r (shorter than r in *yanayacuensis*), and from *V. johnnyrosalesi* by proportion of veins 2RS and r (2.0 × vs 1.4 ×), sculpture of metapleuron and mediotergite 2, and narrower mediotergite 1 (narrowest width 0.6 × width at posterior margin vs 0.8 × in *johnnyrosalesi*).

#### Etymology

*Venanus randallgarciai* is named in honor of Sr. Randall Garcia, currently of San Jose, Costa Rica, but also the first director of ACG and the current Executive Director of the Instituto Nacional de Biodiversidad (INBio), and therefore a major facilitator of ACG biodiversity inventory.

#### Distribution

Only known from a single locality in Area de Conservación Cordillera Volcanica Central, Alajuela, Costa Rica.

#### Notes

We obtained a partial sequence (164 bp) of the DNA barcoding region for the holotype (see also Suppl. material 1). It has the sequence accesion HYCNF533-11 in BOLD (www.boldsystems.org), and the nucleotide sequence is reproduced below:

TTATACCAATTATAATTGGAGGATTTGGAAATTGATTGGTGCCATTAATATTAGGGACTCCAGATATAGCTTTCCCTCGTATAAATAATATAAGATTTTGATTACTTATTCCTTCATTAT TTATATTAATTTTAAGAAGATTCATTAATACAGGCGCAGGTACG

## Identification Keys

### Key to species of *Venanus* from Costa Rica

**Table:** 

1	Metasomal tergite I with slight constriction in width nearer anterior end and very long (at least 2 × as long as apically or basally broad); metasomal tergite II (raised part) rather parallel-sided and close to 2 × as long as broad; propodeum usually with median longitudinal carina pronounced in the anterior third, sometimes percurrently	2
–	Metasomal tergite I without obvious narrowing near anterior end, either posteriorly narrowing throughout, or broadening generally and usually less than 2 × as long as broad; metasomal tergite II less than 2 × as long as broad, variably shaped; propodeum usually without obvious medial longitudinal carina, or with only a stub of one posteriorly or anteriorly	Here it continues to couplet 3 of Whitfield et al. (2011), including all remaining known species of *Venanus*
2	Female body length 2.8–3.0 mm; propodeum with median longitudinal carina marked throughout most of its length, strongly so on anterior 0.3; T2 length 2.5 × its central width [Colombia, Ecuador]	*Venanus helavai* Mason, 1981
–	Female body length at most 2.5 mm (usually less); propodeum with median longitudinally carina only slightly marked on anterior 0.3, or not clearly defined (carina not distinct among other sculpture of propodeum); T2 length less than 1.8 × its central width [Costa Rica, Ecuador]	3
3	Fore wing with first discal cell width 1.0 × its height, and vein 2RS shorter than r; anterior half of propodeum mostly smooth (only with fine rugulosity medially and laterally); metasoma with most laterotergites black [Ecuador, specimens collected at or over 2,100 m]	*Venanus yanayacuensis* Arias-Penna & Whitifield, 2011
–	Fore wing with first discal cell width 1.2 × its height, and vein 2RS significantly longer than r; anterior half of of propodeum as coarsely rugose as posterior half (at most with smooth area partially on anterior 0.1–0.2 of propodeum); metasoma with most laterotergites brown [Costa Rica, specimens collected at 1,400–1,460 m]	4
4	Fore wing with length of vein 2RS 1.4 × length of vein r; metapleuron coarsely sculptured on posterior 0.3 × (in addition of strong impressions on posterior margin); T2 coarsely rugose; T1 relatively less constricted than below, with T1 narrowest width (near anterior margin) 0.8 × width at posterior margin	*Venanus randallgarciai* Fernández-Triana & Whitfield, **sp. n.**
–	Fore wing with length of vein 2RS 2.0 × length of vein r; metapleuron mostly smooth (except for strong impressions on posterior margin); T2 smooth; T1 relatively more constricted than above, with T1 narrowest width (near anterior margin) 0.6 × width at posterior margin	*Venanus johnnyrosalesi* Fernández-Triana & Whitfield, **sp. n.**

Both species from Costa Rica will run through couplet 2 of the key in Whitfield et al. (2011). The key below separates the new species from those mentioned in that couplet.

## Supplementary Material

Supplementary material 1 K2P tree with known sequences of described species of Venanus Data type: DNA barcodes
Brief description: Neighbor-Joining (NJ – Saitou and Nei 1987) tree based on Kimura 2-parameter distances (K2P – Kimura 1980) of all described *Venanus* species with DNA barcodes available. Sequence data from the Barcode of Life Data Systems (http://www.boldsystems.org/). For every sequence the species name, specimen code, length of sequence (in base pairs), and country/province or country/state is shwon.
File: oo_33604.pdf Fernandez-Triana et al. 2014

XML Treatment for Venanus

XML Treatment for Venanus johnnyrosalesi

XML Treatment for Venanus randallgarciai
