# Supplementary material for: First record of the genus Venanus (Hymenoptera: Braconidae: Microgastrinae) in Mesoamerica, with the description of two new species from Costa Rica
Source: Biodivers Data J. 2014 Nov 13;(2):e4167. doi: 10.3897/BDJ.2.e4167 (PMC4238065; doi:10.3897/BDJ.2.e4167)
Supplement: Supplementary material 1 — K2P tree with known sequences of described species of Venanus [file biodiversity_data_journal-2-e4167-s001.pdf]

BOLD TaxonID Tree

Title	:	SEARCH: Process ids(56 ids), Include public records [SEARCH2...
Date	:	31-October-2014
Data Type	:	Nucleotide
Distance Model	:	Kimura 2 Parameter
Marker	:	COI-5P
Codon Positions	:	1st, 2nd, 3rd
Labels	:	Country & Province, SampleID, Sequence Length
Filters	:	Length > 150
Colorization	:	[blue]=Stop Codons [red]=Contamination or misidentification

Sequence Count	:	56
Species count	:	4
Genus count	:	1
Family count	:	1
Unidentified	:	0

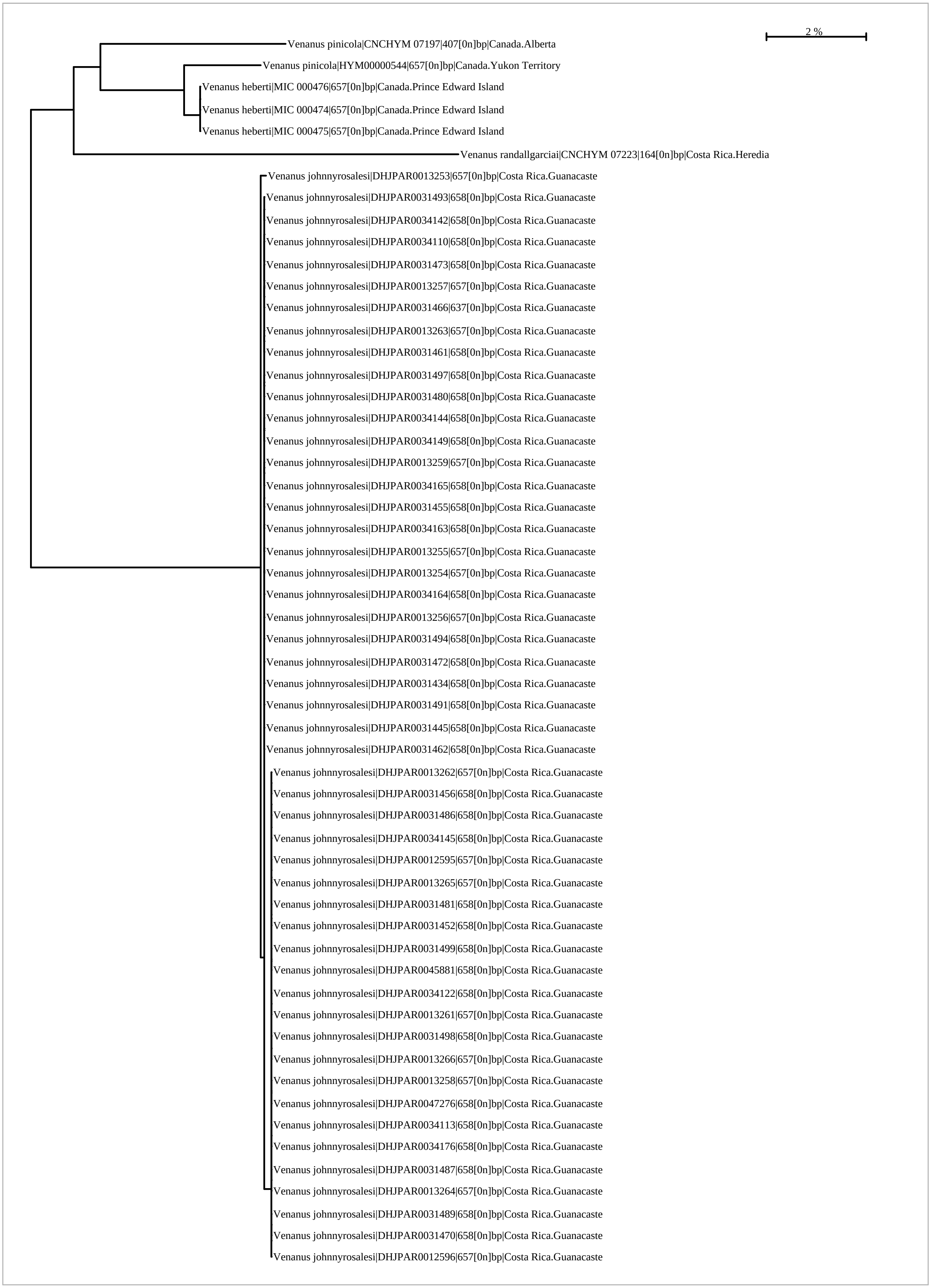
2 %
Venanus pinicola|CNCHYM 07197|407[0n]bp|Canada.Alberta
Venanus pinicola|HYM00000544|657[0n]bp|Canada.Yukon Territory
Venanus heberti|MIC 000476|657[0n]bp|Canada.Prince Edward Island
Venanus heberti|MIC 000474|657[0n]bp|Canada.Prince Edward Island
Venanus heberti|MIC 000475|657[0n]bp|Canada.Prince Edward Island
Venanus randallgarciai|CNCHYM 07223|164[0n]bp|Costa Rica.Heredia
Venanus johnnyrosalesi|DHJPAR0013253|657[0n]bp|Costa Rica.Guanacaste
Venanus johnnyrosalesi|DHJPAR0031493|658[0n]bp|Costa Rica.Guanacaste
Venanus johnnyrosalesi|DHJPAR0034142|658[0n]bp|Costa Rica.Guanacaste
Venanus johnnyrosalesi|DHJPAR0034110|658[0n]bp|Costa Rica.Guanacaste
Venanus johnnyrosalesi|DHJPAR0031473|658[0n]bp|Costa Rica.Guanacaste
Venanus johnnyrosalesi|DHJPAR0013257|657[0n]bp|Costa Rica.Guanacaste
Venanus johnnyrosalesi|DHJPAR0031466|637[0n]bp|Costa Rica.Guanacaste
Venanus johnnyrosalesi|DHJPAR0013263|657[0n]bp|Costa Rica.Guanacaste
Venanus johnnyrosalesi|DHJPAR0031461|658[0n]bp|Costa Rica.Guanacaste
Venanus johnnyrosalesi|DHJPAR0031497|658[0n]bp|Costa Rica.Guanacaste
Venanus johnnyrosalesi|DHJPAR0031480|658[0n]bp|Costa Rica.Guanacaste
Venanus johnnyrosalesi|DHJPAR0034144|658[0n]bp|Costa Rica.Guanacaste
Venanus johnnyrosalesi|DHJPAR0034149|658[0n]bp|Costa Rica.Guanacaste
Venanus johnnyrosalesi|DHJPAR0013259|657[0n]bp|Costa Rica.Guanacaste
Venanus johnnyrosalesi|DHJPAR0034165|658[0n]bp|Costa Rica.Guanacaste
Venanus johnnyrosalesi|DHJPAR0031455|658[0n]bp|Costa Rica.Guanacaste
Venanus johnnyrosalesi|DHJPAR0034163|658[0n]bp|Costa Rica.Guanacaste
Venanus johnnyrosalesi|DHJPAR0013255|657[0n]bp|Costa Rica.Guanacaste
Venanus johnnyrosalesi|DHJPAR0013254|657[0n]bp|Costa Rica.Guanacaste
Venanus johnnyrosalesi|DHJPAR0034164|658[0n]bp|Costa Rica.Guanacaste
Venanus johnnyrosalesi|DHJPAR0013256|657[0n]bp|Costa Rica.Guanacaste
Venanus johnnyrosalesi|DHJPAR0031494|658[0n]bp|Costa Rica.Guanacaste
Venanus johnnyrosalesi|DHJPAR0031472|658[0n]bp|Costa Rica.Guanacaste
Venanus johnnyrosalesi|DHJPAR0031434|658[0n]bp|Costa Rica.Guanacaste
Venanus johnnyrosalesi|DHJPAR0031491|658[0n]bp|Costa Rica.Guanacaste
Venanus johnnyrosalesi|DHJPAR0031445|658[0n]bp|Costa Rica.Guanacaste
Venanus johnnyrosalesi|DHJPAR0031462|658[0n]bp|Costa Rica.Guanacaste
Venanus johnnyrosalesi|DHJPAR0013262|657[0n]bp|Costa Rica.Guanacaste
Venanus johnnyrosalesi|DHJPAR0031456|658[0n]bp|Costa Rica.Guanacaste
Venanus johnnyrosalesi|DHJPAR0031486|658[0n]bp|Costa Rica.Guanacaste
Venanus johnnyrosalesi|DHJPAR0034145|658[0n]bp|Costa Rica.Guanacaste
Venanus johnnyrosalesi|DHJPAR0012595|657[0n]bp|Costa Rica.Guanacaste
Venanus johnnyrosalesi|DHJPAR0013265|657[0n]bp|Costa Rica.Guanacaste
Venanus johnnyrosalesi|DHJPAR0031481|658[0n]bp|Costa Rica.Guanacaste
Venanus johnnyrosalesi|DHJPAR0031452|658[0n]bp|Costa Rica.Guanacaste
Venanus johnnyrosalesi|DHJPAR0031499|658[0n]bp|Costa Rica.Guanacaste
Venanus johnnyrosalesi|DHJPAR0045881|658[0n]bp|Costa Rica.Guanacaste
Venanus johnnyrosalesi|DHJPAR0034122|658[0n]bp|Costa Rica.Guanacaste
Venanus johnnyrosalesi|DHJPAR0013261|657[0n]bp|Costa Rica.Guanacaste
Venanus johnnyrosalesi|DHJPAR0031498|658[0n]bp|Costa Rica.Guanacaste
Venanus johnnyrosalesi|DHJPAR0013266|657[0n]bp|Costa Rica.Guanacaste
Venanus johnnyrosalesi|DHJPAR0013258|657[0n]bp|Costa Rica.Guanacaste
Venanus johnnyrosalesi|DHJPAR0047276|658[0n]bp|Costa Rica.Guanacaste
Venanus johnnyrosalesi|DHJPAR0034113|658[0n]bp|Costa Rica.Guanacaste
Venanus johnnyrosalesi|DHJPAR0034176|658[0n]bp|Costa Rica.Guanacaste
Venanus johnnyrosalesi|DHJPAR0031487|658[0n]bp|Costa Rica.Guanacaste
Venanus johnnyrosalesi|DHJPAR0013264|657[0n]bp|Costa Rica.Guanacaste
Venanus johnnyrosalesi|DHJPAR0031489|658[0n]bp|Costa Rica.Guanacaste
Venanus johnnyrosalesi|DHJPAR0031470|658[0n]bp|Costa Rica.Guanacaste
Venanus johnnyrosalesi|DHJPAR0012596|657[0n]bp|Costa Rica.Guanacaste
